# Acupuncture for the treatment of phantom limb pain in lower limb amputees: study protocol for a randomized controlled feasibility trial

**DOI:** 10.1186/s13063-015-0668-3

**Published:** 2015-04-12

**Authors:** Esmé G Trevelyan, Warren A Turner, Nicola Robinson

**Affiliations:** Faculty of Health and Social Care, London South Bank University, 103 Borough Road, London, SE1 0AA UK

**Keywords:** Acupuncture, feasibility studies, pain, phantom limb

## Abstract

**Background:**

Phantom limb pain is a prevalent condition that is difficult to manage, with a lack of robust evidence to support the use of many adjunctive treatments. Acupuncture can be effective in the management of many painful conditions but little is known about its effectiveness in treating phantom limb pain. The aim of this study is to explore the feasibility of conducting a randomized controlled trial comparing acupuncture and routine care in a group of lower limb amputees with phantom limb pain.

**Methods/design:**

An unstratified, pragmatic, randomized, two-armed, controlled trial of parallel design comparing acupuncture and usual care control will be conducted. A total of 20 participants will be randomly assigned to receive either usual care or usual care plus acupuncture. Acupuncture will include eight 1 hour treatments delivered pragmatically over 4 weeks by practitioners trained in traditional Chinese medicine. As outcome measures, the Numerical Pain Rating Scale, short-form McGill Pain Questionnaire 2, EQ-5D-5 L, Hospital Anxiety and Depression Scale, 10-Item Perceived Stress Scale, Insomnia Severity Index, and Patient Global Impression of Change will be completed at baseline, weekly for the duration of the study and at 1 month after completion of the study. After completion of the trial, participants will provide feedback though semi-structured interviews.

Feasibility will be determined through the ability to recruit to the study, success of the randomization process, completion of acupuncture intervention, acceptability of random allocation and completion of outcome measures. Acceptability of the acupuncture intervention will be determined through semi-structured interviews with participants. The appropriateness of outcome measures for a future trial will be addressed through completion rates of questionnaires and participant feedback.

**Discussion:**

Data generated on effect size will be used for future sample size calculations and will inform the development of an appropriate and feasible protocol for use in a definitive multicentre randomized controlled trial.

**Trial registration:**

ClinicalTrials.gov: NCT02126436.

**Electronic supplementary material:**

The online version of this article (doi:10.1186/s13063-015-0668-3) contains supplementary material, which is available to authorized users.

## Background

Phantom limb pain, defined as painful sensations perceived in the missing portion of the amputated limb [1], was recorded medically as early as the sixteenth century by Ambroise Paré [2]. It is very common and prevalence may be as high as 75 to 80% [3,4].

Treatment of phantom limb pain includes such interventions as pre-emptive analgesia, pharmacological interventions, neuromodulation and supportive nonpharmacological or noninvasive techniques, such as mirror therapy, graded motor imagery and stump liners. Evidence suggests that pre-emptive epidural and perineural analgesia might not prevent chronic phantom limb pain [5,6]. Pre-emptive gabapentin has also been found to be ineffective [7]. Pharmacological interventions, including morphine, gabapentin and ketamine, may provide short term analgesic efficacy [8]. There is a lack of robust evidence to support the use of neuromodulation [9], mirror therapy [10] or graded motor imagery [11]. One randomized controlled trial suggested that stump liners might reduce phantom limb pain [12] but the sample size in this study was small.

Acupuncture has been shown to be an effective intervention in the management of many pain conditions [13-15] but little is known about the effectiveness of acupuncture for the treatment of neuropathic pain [16]. Specifically, the effectiveness of acupuncture for treating phantom limb pain has not been widely assessed or documented, with most of the literature consisting of case reports [17,18]. Although these studies generally report positive outcomes [17], they are at the bottom of the hierarchy of evidence [19]. A systematic review including English, Chinese and Korean databases identified only two nonrandomized controlled trials evaluating the effectiveness of acupuncture. Although these studies reported positive outcomes, both were deemed to have a high risk of bias and low methodological quality [20]. Further research is needed to evaluate the effectiveness of acupuncture for treating phantom limb pain but, prior to a definitive trial, a study is needed to determine feasibility [21].

The objectives of this study are to: (1) explore the feasibility of recruiting, randomizing and retaining participants; (2) evaluate the feasibility and acceptability of including a standard care control; (3) evaluate the adherence or compliance and acceptability of acupuncture as an intervention; (4) evaluate the appropriateness of outcome measures and their completion rates and explore participants’ experience in completing outcome measures; (5) identify appropriate primary and secondary outcome measures that could be used in future trials; (6) explore the perceived effectiveness of acupuncture in treating phantom limb syndrome; (7) generate data on effect size for use in future sample size calculations; and (8) inform the development of an appropriate and feasible protocol for use in a definitive multicentre randomized controlled trial.

## Methods/design

### Design

A comparative effectiveness feasibility study will be conducted, using a mixed-methods approach, including a small randomized controlled trial and semi-structured interviews. The randomized controlled trial will be an unstratified, open, pragmatic, effectiveness trial, of parallel design, with two arms, using balanced randomization between acupuncture and usual care and a usual care control. Cross-sectional interviews will be carried out at the end of the intervention period.

Ethical approval was granted from the National Research Ethics Service Committee London (Bloomsbury) in July 2014 and Guy’s and St Thomas’ R & D and London South Bank University in October 2014. The trial is registered with ClinicalTrials.gov (NCT02126436) https://www.clinicaltrials.gov/ct2/show/study/NCT02126436 and will be conducted in compliance with the principles of the Declaration of Helsinki [22] the London South Bank University Code of Practice, and the London (Bloomsbury) Research Ethics Committee.

### Study settings

The study will be conducted at the Amputee Rehabilitation Unit, at Lambeth Community Care Centre, London, Guy’s and St Thomas’ NHS Foundation Trust. The Amputee Rehabilitation Unit is a 12-bed inpatient unit that provides specialist rehabilitation after major amputation. It accepts both primary and established amputees who have undergone a functional decline for approximately 7 weeks of evidence-based care, including access to specialist medical, nursing, therapy, and counselling professionals. Acupuncture will be provided at the Gateway Acupuncture Clinic (which is located in the same building) and provides an NHS acupuncture service through GP referral, in the area of Lambeth and Southwark. The clinic provides treatment for chronic long-term conditions, specializing in chronic pain, headaches, migraines and HIV.

### Recruitment

**Figure 1:**
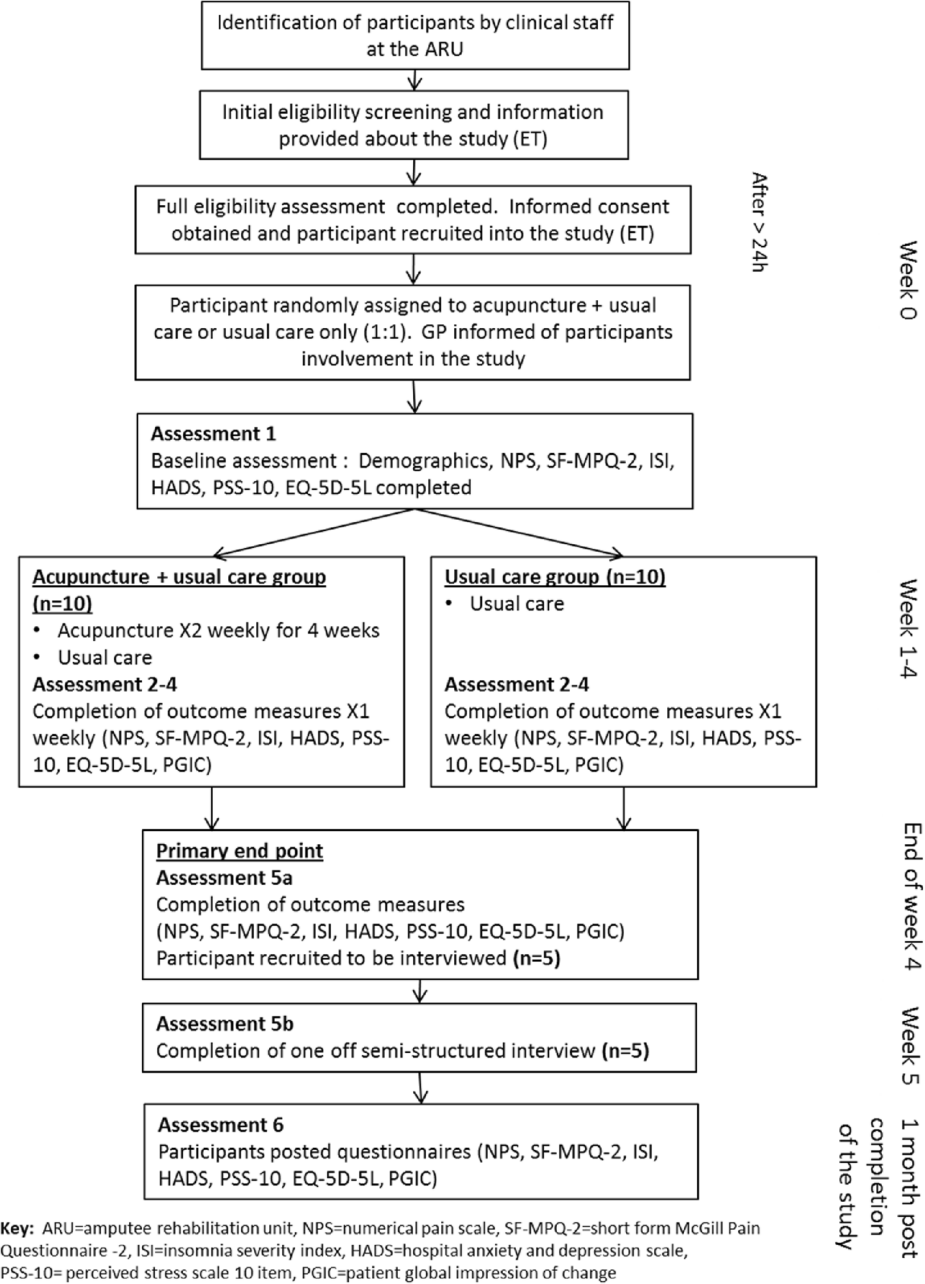
Participants’ flow through the study. The different phases of the study and participants’ flow through the study with details on timings of the different phases of the study. ARU, Amputee Rehabilitation Unit; GP, general practitioner; HADS, Hospital Anxiety and Depression Scale; ISI, Insomnia Severity Index; NPS, numerical pain scale; PGIC, Patient Global Impression of Change; PSS-10, 10-Item Perceived Stress Scale; SF-MPQ-2, short-form McGill Pain Questionnaire 2.

Participants will be approached and recruited whilst they are inpatients at the Amputee Rehabilitation Unit. Newly admitted potential participants will be identified by clinical staff (JG, CC), approached by the researcher (EGT), initially screened, and provided with oral and written information. Participants who pass initial screening tests and are willing to participate will have a final eligibility check by EGT before enrolment in the study (Figure 1). Signed, informed consent will be obtained from all participants before enrolling them in the study. Those declining to participate will be asked briefly for their reasons. As the study is a feasibility study, no sample size calculation has been performed [21]. An arbitrary number of 20 participants was deemed adequate to provide information on recruitment, randomization and acceptability of acupuncture and answer the objectives of this trial [23].

### Eligibility criteria

Participants will be included if they: (1) at least 18 years of age, (2) have full cognitive ability and are able to communicate in English, (3) have traumatic or medical amputation of a lower limb (greater than toes), and (4) are currently experiencing phantom limb pain of ≥5 on an 11-point verbal rating scale (that is, moderate or severe phantom limb pain) [24]. Participants will be excluded if they: (1) have congenital limb absence, (2) are medically too unwell (as advised by medical staff at the Amputee Rehabilitation Unit), (3) are pregnant, or (4) where acupuncture is cautioned, including participants with poorly controlled epilepsy, severe haemophilia or other bleeding or clotting disorders, or a pacemaker (if using electro-acupuncture), and patients undergoing or who have recently undergone chemotherapy or bone marrow transplant, any skin changes or removal of lymph nodes in the body, that would preclude placement of acupuncture needles [25].

### Intervention (RCT)

The acupuncture group (group A) will receive usual inpatient care and a course of acupuncture. The control group (group C) will receive usual inpatient care only. Usual care will include both medical intervention and daily physiotherapy and rehabilitation, as routinely provided at the Amputee Rehabilitation Unit.

All acupuncture practitioners involved in the study will be trained in the practice of traditional Chinese medicine. All practitioners will be members of the British Acupuncture Council, will have at least 15 years clinical experience and will follow the safety guidelines of the British Acupuncture Guide to Safe Practice [25]. Acupuncture needles will be single-use, presterilized, disposable, solid, stainless steel needles (with prepacked single-use guide tubes if guide tubes are used). The acupuncture intervention will be pragmatic but will be guided by a protocol previously developed though a Delphi practitioner consensus study [26]. This protocol advises:

- Using a combination of body and auricular acupuncture;
- Treating the contralateral limb and possibly the ipsilateral limb;
- Including auricular acupuncture points such as shen men, sympathetic and points corresponding to the lower limb;
- Depending on the health of the tissue and the individual participant, needling around the stump;
- Mirroring local and distal points by needling the opposite limb;
- Including points on the lower back (taking a segmental approach to dermatomal pain);
- Including points such as LI4 + LR3, LR3, GV20, SP10 and also specified points according to participants’ specific symptoms;
- Retaining needles for 20 to 30 minutes.

No set criteria will be followed when needling, other than to follow the guidelines of the protocol. Practitioners will assess and treat participants under the paradigm of traditional Chinese medicine. Treatment may (depending on the practitioner’s diagnosis and treatment plan) include electro-acupuncture or other adjunctive interventions, such as cupping. All participants in group A will receive eight one-hour acupuncture sessions (twice weekly for 4 weeks) delivered during their inpatient stay.

### Withdrawals, discontinuation and post-trial care

Acupuncture is a low-risk intervention and any adverse effects are usually minimal and temporary. In the event of mild adverse effects (drowsiness, haematoma, bleeding from a point, stuck needle, pain after needling a point) participants will not be withdrawn but will be able to drop out if they so wish. In the event of the occurrence of more serious adverse events, participants will be withdrawn. As evidence suggests that acupuncture is a safe treatment [27,28] and as this study is not assessing effectiveness, no specific arrangements have been made to review interim safety and effectiveness. However, in the event of three participants reporting prolonged aggravation of pain or other potential serious adverse events, the trial will be stopped prematurely. After completion of the study, participants will be offered access to acupuncture through their general practitioner or physiotherapist.

### Concomitant care

Participants will be asked to refrain from using other forms of complementary therapy for the duration of the trial but may receive any intervention as routinely prescribed by clinical staff at the Amputee Rehabilitation Unit.

### Study restrictions

Participants and practitioners will be asked not to disclose participant allocation to the researcher (EGT).

### Intervention (interviews)

Consecutive sampling will be used to recruit (*n* = 5) participants from group A to explore their experience of being in the trial, having acupuncture and completing outcome measures. Participants will be interviewed once after completion of the study. Semi-structured interviews will be facilitated by EGT, will follow a topic guide (Additional file 1) and will be recorded and transcribed verbatim.

### Randomization, allocation concealment and blinding

Randomization and allocation concealment will be used to ensure against selection bias [29]. Prior to commencement of the study, a researcher (NR) not involved in the day-to-day execution of the study will randomly allocate and conceal allocation. A copy of the randomized sequence will be kept in a locked cabinet and not shared with study personnel. The researcher (EGT) who will enrol participants and assign them to either acupuncture intervention or control will not know the random sequence or treatment allocation.

Randomization will be achieved using a computer-generated random numbers table and will be unstratified and balanced (1:1). Permuted blocking will be used to achieve balance between study arms. A block size of four will be used in this study. Allocation concealment will be implemented using sequentially numbered, opaque sealed envelopes. The envelopes will be opened sequentially only after participant’s details are written on each envelope.

Participants and practitioners involved in the study will not be blinded. However, the researcher collecting outcome measures (EGT) will be blind to the participant’s allocation.

### Outcome measures

Recommendations from the Assessment Committee of the Neuropathic Pain Special Interest Group [30] and the Initiative on Methods, Measurements, and Pain Assessment in Clinical Trials [31] were taken into consideration when developing outcome measures for use in this trial.

The primary outcome measure will be an 11-point numerical rating scale to record pain intensity. Pain will be rated by a number describing average pain over the previous week, using the anchors 0, meaning ‘no pain’, and 10, meaning ‘pain as bad as you can imagine’ [31]. The numerical rating scale is an appropriate measure of pain intensity [32] and is a recommended outcome measure for clinical trials of chronic pain treatment effectiveness [31].

Secondary outcome measures will include a numerical rating scale measuring ‘worst’ pain, the short-form McGill Pain Questionnaire 2 (SF-MPQ-2), EQ-5D-5 L, Hospital Anxiety and Depression Scale, 10-Item Perceived Stress Scale, Insomnia Severity Index, and Patient Global Impression of Change.

The McGill Pain Questionnaire and its short form (SF-MPQ) are generic questionnaires (applicable to any pain), whose reliability and validity have been extensively documented [33]. The SF-MPQ-2 is a 22-item questionnaire that uses a 10 point rating scale and records the major symptoms of both neuropathic and non-neuropathic pain [34]. It is reliable and valid for measuring diverse chronic pain [35].

The EQ-5D measures health-related quality of life and although not validated for use in neuropathic trials, results appear robust in neuropathic trials with a large sample size or when recording a large-pain relief response in the active group [30]. The EQ-5D-5 L has the same core dimensions as the EQ-5D but instead of a three-point rating scale it uses five levels.

The Hospital Anxiety and Depression Scale measures emotional function and is responsive to change in neuropathic pain clinical trials [30]. It includes seven depression and seven anxiety items to cover cognitive and emotional aspects of depression and anxiety and is reliable and valid for assessing emotional distress in a medical population [36].

The Perceived Stress Scale is a 4-, 10- or 14-item questionnaire that was designed to measure psychological stress [37]. It is reliable and valid and was found to have acceptable psychometric properties across 19 studies [38]. The 10-item version has no loss of psychometric quality, compared with the 14-item version [39] and has, in fact, been shown to be superior [38].

The Insomnia Severity Index measures perception of insomnia and the degree of concerns or distress caused by insomnia; it consists of seven items [40]. It has the advantage of measuring symptoms over a 2-week period and is recommended when insomnia is a secondary endpoint. It has been validated against both polysomnographic and prospective sleep diary measures [40,41].

The Patient Global Impression of Change scale is advised for use in clinical trials [30,31] and provides a readily interpretable assessment of participants evaluation of the importance of their improvement [42]. The scale used in this study will be a seven-point scale that ranges from ‘no change or worse’ to ‘a great deal better’ [43].

### Assessment

Participants will complete the questionnaires and rating scales six times in total. Data for all items (except the Patient Global Impression of Change, data for which will only be collected from the end of week one) will be collected at the time of enrolment and at the end of each week for the duration of the study. The primary endpoint will be at the end of the intervention (end of week 4). Data for the outcome measures will also be determined one month after completion of the study. Questionnaires and rating scales will be completed under the supervision of the researcher (EGT) at baseline and during weeks 1 to 4 (whilst participants are inpatients at the Amputee Rehabilitation Unit), and will be posted to participants one month after completion of the study.

### Analysis

As this is a feasibility study, emphasis will be on feasibility and not on statistical significance of results [21]. Compliance with the protocol will be examined through number counts on drop outs or numbers of missed treatments, completion rates of outcome measures, and their perceived appropriateness and deviation from the protocol. Data will be collected on the use of rescue medication and adverse events (captured through open-ended prompts by practitioners at each intervention time point). Details of any participants who are excluded from the study will be reported and exclusion will be distinguished from attrition.

All statistical analysis will be undertaken using SPSS Version 21 software. The analysis will test for within-patient and between-group differences in measurements taken at the beginning of the study, during the study, at the end of the study and one month after completion of the study. An intention-to-treat approach will be taken [44]. To include missing data, any missing data will be imputed using the last observation carried forward [45].

The null hypothesis is that there is no difference in change in the primary outcome measure between group A and C at the end of intervention (end of week 4). Statistical analysis will be performed to verify rejection of the null hypothesis with a *P* = 0.05 taken as indicative of statistical significance. Nonparametric tests will be used in inferential analysis. The nonparametric Mann–Whitney *U* test will be used for analysis between groups. The difference between baseline and last observation scores will be analyzed using Wilcoxon signed-rank test. The effect size (Cohen’s *d*) will be calculated to provide information on the relative magnitude of difference [46]. All secondary outcome measures will be treated in the same way as the primary outcome. Categorical and continuous baseline characteristics will also be analyzed to test for between-group differences.

A framework analysis procedure [47] will be used to analyze qualitative data. NVivo 10 software will be used to develop the analytic framework and index transcripts. Microsoft Excel will be used during charting. Interviews will be transcribed verbatim. Specific steps will be followed during data analysis, including; familiarization, coding, identifying an analytic framework, indexing, charting and mapping or interpretation. To ensure credibility, peer debriefing will take place throughout the research process. To ensure dependability, two researchers will separately code a selection of transcripts.

The trial will be considered successful if:

- Recruitment rate is ≥2 participants per month fitting the eligibility criteria.
- The study recruits ≥70% of all eligible potential participants.
- Of the participants recruited to group A, ≥90% receive their first acupuncture treatment within one week of recruitment.
- After randomization and allocation, ≥90% of participants receive treatment as initially intended.
- Of the participants recruited to group A, ≥80% receive all eight acupuncture treatments.
- Of the participants recruited to group C, ≤10% drop out of the study.
- At the primary endpoint of the study, questionnaires and rating scales for outcome measures are completed by ≥90% of participants.
- At one month after completion of the study, questionnaires and rating scales for outcome measures are completed by ≥60% of participants.
- Qualitative data identifies that outcome measures are acceptable and appropriate, that questionnaires and rating scales are easy to complete and that outcome measures can be identified for used in a definitive trial.
- Qualitative data implies that acupuncture is an acceptable and effective intervention for treating phantom limb pain with or without other secondary symptoms.
- Qualitative and quantitative data implies that the acupuncture protocol used in the feasibility study is appropriate for use in a definitive multicentre randomized controlled trial.

### Data management and reporting

The Consolidated Standards of Reporting Trials (CONSORT) [48] and Standards for Reporting Interventions in Clinical Trials of Acupuncture (STRICTA) [49] will be adhered to when reporting. Data will be stored securely with no participant identifiers included. Access to and handling of data will be restricted to those involved in the study. All disseminated findings will contain no participant-identifiable data.

## Discussion

Currently, no genuine placebo-controlled acupuncture trials exist [50]. Different types of sham acupuncture have been implemented in acupuncture trials, including; shallow needling of acupuncture points, using nonpenetrating needles, needling non-acupuncture points and needling acupuncture points that are not indicated for that specific condition, but none of these methods is physiologically inert [50] and sham acupuncture might have some level of effectiveness [51]. Therefore, despite a lack of blinding introducing ascertainment bias, sham acupuncture will not be used in this study and will not be used in a future definitive trial.

Inferential statistical analysis and hypothesis testing will be completed only to pilot procedure and will not be interpreted as a measure of effectiveness of acupuncture. Effectiveness will only be established after completion of a fully powered randomized controlled trail.

Results of this study will be disseminated through publication.

## Trial status

Recruitment commenced in October 2014 and it is anticipated that it will finish by October 2015.

## Additional file

Additional file 1:
**Summary of the interview topic guide.** This guide will be used to interview participants in the acupuncture group after completion of the randomized controlled trial. PLP, phantom limb pain.

## Abbreviation

SF-MPQ-2: short-form McGill Pain Questionnaire 2
