# Supplementary figures and images for: Acupuncture for the treatment of phantom limb pain in lower limb amputees: study protocol for a randomized controlled feasibility trial
Source: Trials. 2015 Apr 12;16:158. doi: 10.1186/s13063-015-0668-3 (PMC4405855; doi:10.1186/s13063-015-0668-3)

**Additional file 1. Summary of the interview topic guide**


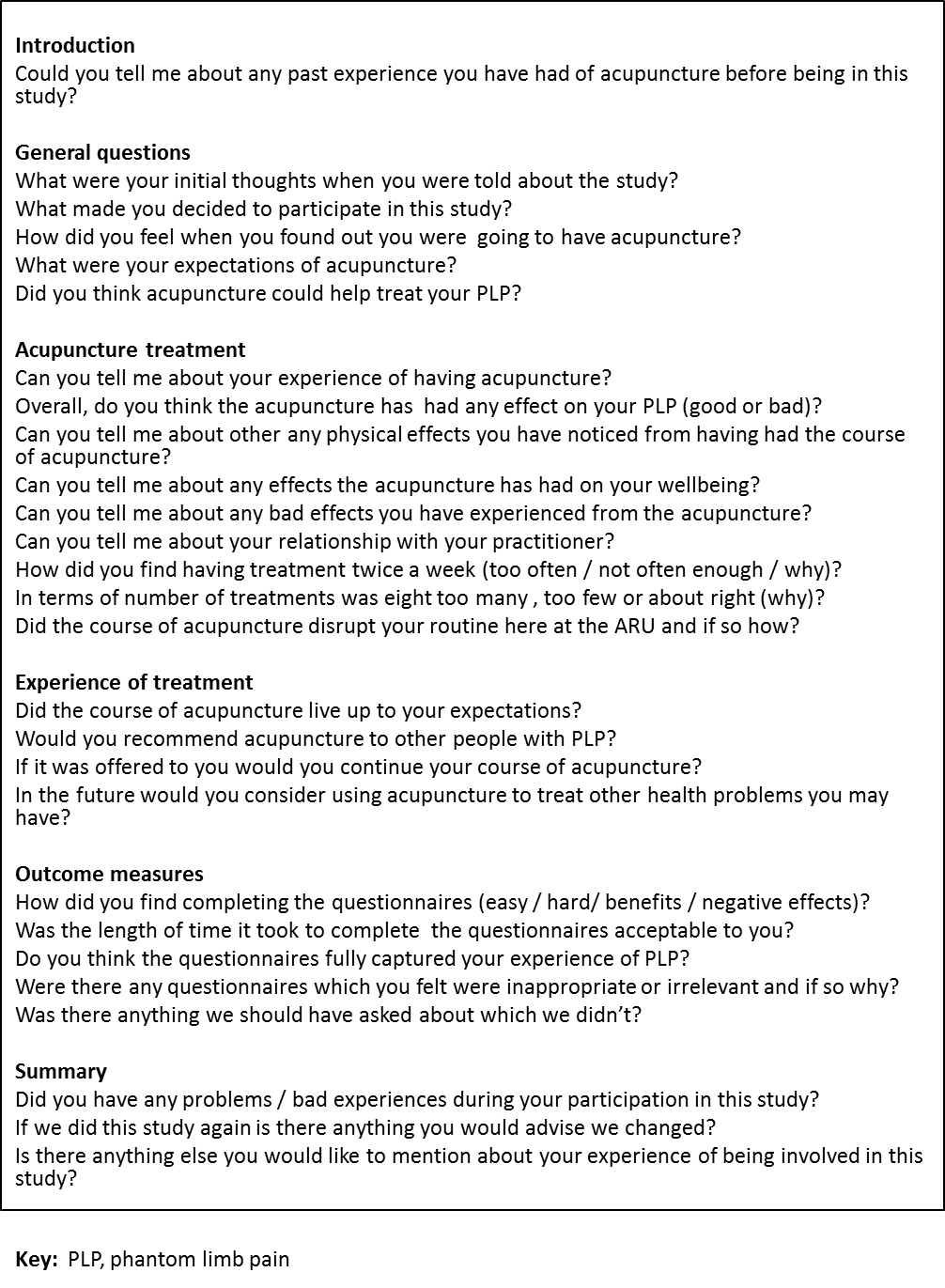

Supplement: Additional file 1: — Summary of the interview topic guide. This guide will be used to interview participants in the acupuncture group after completion of the randomized controlled trial. PLP, phantom limb pain. [file 13063_2015_668_MOESM1_ESM.docx]
